# Socio-demographic characteristics and outcomes of pregnant women who delivered prior to and after the termination of the one-child policy in China: a comparative study

**DOI:** 10.1186/s12884-021-03740-6

**Published:** 2021-04-21

**Authors:** Xiaohui Zhang, Haifeng Lou, Xuejuan Tang, Xiaoli Chen

**Affiliations:** 1 Department of women’s health, Women’s Hospital, School of Medicine, Zhejiang University, Xueshi Road 1, Hangzhou Zhejiang, 310006 P.R. China; 2 Women’s Hospital, School of Medicine, Zhejiang University, Xueshi Road 1, Hangzhou Zhejiang, 310006 P.R. China; 3 Department of women’s health, Jiaxing Maternal and Child Health Hospital, Jia Xing, Zhonghuan Eastern Road, 2468 Zhejiang, P.R. China; 4 Department of pathology, Women’s Hospital, School of Medicine, Zhejiang University, Xueshi Road 1, Hangzhou Zhejiang, 310006 P.R. China

**Keywords:** Fertility policy, Pregnancy outcomes, Cesarean section

## Abstract

**Background:**

The new Chinese fertility policy has recently received widespread public attention. However, there are few studies available on the comprehensive epidemiology of maternal and infant health with respect to the characteristic changes of childbearing women. In the study, we compared the maternal characteristics and pregnancy outcomes at different time points according to policy adjustments, accessed the possible relationship among these factors, and evaluated the impacts of these policies for medical and policy assistance.

**Methods:**

This was a retrospective study. Data were collected from three representative hospitals in Zhejiang Province using stratified random sampling. The annual number of births, and maternal and child healthcare levels were the determining factors of sampling. Women who gave birth in November of 2012, 2014, and 2016 were recruited in accordance with the time of the change in the fertility policy, and we explored the differences in maternal socio-demographic characteristics, delivery mode and pregnancy outcomes.

**Results:**

A total of 11,718 women were recruited, including 3480, 4044, and 4194 in November of 2012, 2014, and 2016, respectively. The proportions of multiparous women, women who aged ≥35 years, who received higher education, who had previous cesarean sections (CS), and who delivered in a high level hospital increased over time. In 2016, multipara accounted for 49.12, 14.47% were aged ≥35 years, nearly half of women had previous CS and delivered in a provincial hospital, 41.73% gave birth by CS, and 31.62% suffered pregnancy complications. The results of multiple logistic regression mode showed CS risk decreased significantly in 2014 (OR_adj_ = 0.62; 95% CI, 0.55–0.67) relative to 2012, and risks of pregnancy complications (OR_adj_ = 2.30; 95% CI, 1.86–2.83) and multiple births (OR_adj_ = 3.25; 95% CI, 2.19–4.83) only increased in 2016 compared to 2012.

**Conclusions:**

Some pregnancy outcomes increased as several key characteristics of childbearing women changed after China ended its “one-child” policy. This suggests that policy providers and medical staff need to strengthen healthcare in a consistent fashion regarding changes in birth policy.

## Background

China has the largest population of any country in the world. A national “one-child” family planning policy was implemented in 1979 to balance the sudden increase in population with economic stagnation [1, 2]. However, with rapid socioeconomic development, China is confronted with enormous challenges from fertility decline, an aging workforce, and other related problems. Therefore, in November 2013, the Chinese government terminated the “one-child” policy, which was then replaced by a “second-child” policy. With respect to the new policy, couples were encouraged to have two babies if one parent was the only child; however, the implementation of the “second-child” policy did not result in the expected surge in population over a relatively brief period [2, 3]. As a result, it was declared in October 2015 that all couples in China would be allowed to have two children as of 2016 [2]. This indicated the initiation of the “universal second-child” policy.

Replacing the “one-child” policy with the “universal second-child” policy has received widespread public attention. Although the new policy provided all couples with equal birth privileges, this renewed reproductive awareness in China heightened social polarization. Accordingly, some researchers have focused on the potential changes associated with population size, health, sex ratio, and natural and environmental resources [3–5]. There are few published studies on the comprehensive epidemiology of maternal and infant health concerning the characteristic changes that occur in childbearing women. Zhejiang Province, which is located on the eastern coast of China and which possesses a well-developed economy, has established the new fertility policy. A review of clinical records has shown an increase in the proportions of pregnant women of advanced reproductive age, and of women undergoing repeated cesarean deliveries. In this report, we compared the maternal characteristics and pregnancy outcomes at different time points according to policy adjustments, accessed the possible differences among these factors that could be associated with the changed birth policy. The findings would be beneficial for medical and policy assistance.

## Methods

### Antenatal health care

In Zhejiang Province, all women are routinely provided with at least five sessions of antenatal health care during pregnancy, according to national guidelines. Herein, pregnant women attended their first antenatal health care session in the first trimester (before 13 weeks), and they were offered the second and the third antenatal health care during their second trimester (suggested at 14–19 weeks and 20–24 weeks, respectively). They were then required to have two antenatal care sessions during the third trimester (≥28 weeks). If identified with pregnancy complications, more antenatal health care was recommended throughout the entire pregnancy, or women were transferred to the corresponding hospital to ensure maternal and infant safety.

### Study design and study population

The current study is a retrospective analysis based on routine medical records. We selected three hospitals and decided on the specific hospitals by stratified random sampling. The annual number of births and maternal and child healthcare levels were the determining factors of sampling. The institutions involved were a university hospital (Women’s Hospital of Zhejiang University), a municipal hospital (Jiaxing Maternal and Child Health Hospital), and a community hospital (Haining Maternal and Child Health Hospital)—representing tertiary, secondary, and primary maternal and child healthcare, respectively. All women who gave birth in the three hospitals in November of 2012, 2014, or 2016 were included, regardless of their pregnancy outcomes and whether being transferred from other hospitals for pregnancy high risk factors. The study times chosen were in accordance with the actual periods of changes in fertility policy.

**Fig. 1:**
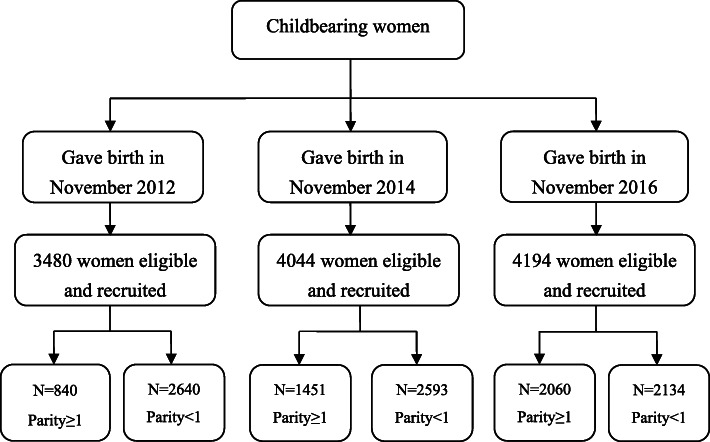
The flow diagram for the inclusion process

The sample size was calculated by the following formula,

$$ N=\frac{{Z^2}_{\alpha /2}\sum {N_i}^2{P}_i\left(1-{P}_i\right)/{W}_i}{N^2{d}^2+{Z^2}_{\alpha /2}\sum {N}_i{P}_i\left(1-{P}_i\right)}, $$

where N is the sample capacity, *α* is test level (0.05), *d* is the permissible deviation (0.03), *P*_*i*_ is the estimated probability value,and *W*_*i*_ is the percentage for each level. In our study, the cesarean section (CS) rate was used to estimate sample size because it was heavily affected by birth policies in China, and was also a factor of concern for pregnant women. The average CS rate was regarded as *P*_*i*_, which was 0.4, and *W*_*i*_ was equal to 0.5, 0.3, and 0.2 from the tertiary, secondary, and primary care levels, respectively. According to the formula above, the sample size in tertiary, secondary, and primary care hospitals should be at least 1000, 600, and 400 individuals, respectively. Thus, our sample size for each hospital appeared to be reasonable to continue analysis. The inclusion process and actual number of the included subjects are shown in Fig. 1.

### Data collection and definition

In the study, we used secondary data derived from hospital electronic medical records, local maternal and child healthcare system, as well as maternal and child healthcare handbooks in Zhejiang Province. Information involved in maternal demographic characteristics, gravidity, parity, pregnancy complications, delivery mode, primary indications for CS, and maternal and neonatal health outcomes. Investigators (specifically trained medical staff) in each participating hospital were responsible for data collection, data entry, and quality control at their own hospital. Finally, all data were sent to the Women’s Hospital, School of Medicine, Zhejiang University, where researchers conducted a second round of quality control assessment, summarization, and analysis. The appointed staff first examined the materials to ensure completeness; second, data were inputted into Epidata 3.1 by two individuals, which provided a logic check. Finally, we continued to inquire about outliers or missing data from local hospitals.

Women with a pre-pregnancy body mass index (BMI) ≥25 kg/m^2^ were categorized into overweight and obese groups. Abortion history indicated that women underwent an artificial abortion during a previous pregnancy. CS after the onset of labor was defined as an emergency CS—primarily due to failure to progress, fetal distress, or intrapartum hemorrhage. Elective CS was performed before the onset of labor for obstetric indications or at the mother’s request. Preterm delivery was defined as birth < 37 weeks of gestation, low birth weight (LBW) was assigned to a birth weight under 2500 g, and stillbirth was defined as fetal death occurring at 28 weeks of gestation or later. Pregnancy complications were diagnosed by a physician and included premature rupture of membranes, gestational hypertension, gestational diabetic mellitus, and severe anemia and others. Birth defects (BDs) indicated anomalies identified in fetus or infants within 7 days after birth in hospital.

### Statistical analysis

We inputted data from this study into Epidata 3.1 and analyzed them using SPSS for Windows software, version 20.0. Categorical data are presented as frequencies and percentages, and quantitative data are presented as mean ± standard deviation (SD). A socio-demographics distribution change with time was compared by Chi-squared test. The crude odds ratios (OR) and 95% confidence intervals (CI) were estimated to analyze the correlations among maternal age, delivery mode, and pregnancy outcomes before and after ending the one-child policy. Data in 2012 were regarded as the corresponding reference group. OR 1 signified the comparison 2014/2012 and OR 2 signified 2016/2012. Considering the potential confounders, we performed stratified analysis by maternal parity since this index might be heavily impacted by policy changes. After adjusting the confounders, we used multiple logistic regression analysis to explore the relationship between these factors and impacts of policy changes. *P* < 0.05 was considered statistically significant.

## Results

### Socio-demographic characteristics

A total of 11,718 women gave birth, including 3480 in 2012, 4044 in 2014, and 4194 in 2016. The mean age of the childbearing women was 28.80 ± 4.72 years, ranging from 15 to 55, and maternal socio-demographic characteristics were obviously different over time. During the period analyzed, the proportions of multipara, women ≥35 years of age, those who received at least a secondary education, those who showed gravidity ≥2, those who had a previous CS, and those who delivered a baby in a provincial hospital increased, but the proportions of women who were overweight or obese and with an abortion history diminished (Table 1).

**Table 1:** Comparison of socio-demographic characteristics

Variable		2012 (***N*** = 3480)		2014 (***N*** = 4044)		2016 (***N*** = 4194)		*x*^2^	***P***
		***n***	%	***n***	%	***n***	%		
Primipara		2640	76.86	2593	64.12	2134	50.89	512.52	< 0.001
Multipara		840	24.14	1451	35.88	2060	49.12		
Maternal age ≥ 35		228	6.55	355	8.78	607	14.47	143.63	< 0.001
For primipara ≥35		78	2.95	75	2.89	85	3.98	25.04	< 0.001
For multipara ≥35		150	17.86	278	19.16	522	25.34	28.91	< 0.001
Education	**≥**Secondary	2380	70.50	2934	74.64	3051	75.74	28.35	< 0.001
	Missing	104	–	113	–	166	–		
Gravidity	≥2	1803	51.81	2252	55.69	2672	63.71	117.61	< 0.001
Overweight or obese		2390	68.67	2752	68.07	2189	52.19	300.11	< 0.001
Previous CS		380	25.03	688	42.47	868	49.60	132.89	< 0.001
Abortion history		1363	39.17	1377	34.05	1465	34.93	23.86	< 0.001
Delivery hospital	Provincial	1276	36.67	1625	40.18	2021	48.19	192.55	< 0.001
	Municipal	1408	40.46	1557	38.50	1637	39.03		
	Community	796	22.87	862	21.32	536	12.78		

### Mode of delivery and pregnancy outcomes

Table 2 displays the crude comparison of delivery mode and pregnancy outcomes after the policy changes. For all childbearing women, a decreasing OR for overall CS was observed between 2012 and 2014. Compared with 2012, the rate of instrumental vaginal birth and emergency CS increased in 2014; however, the rate of emergency CS decreased significantly in 2016. Both the OR for stillbirth and BDs decreased over time. A series of increasing OR values for pregnancy complications, multiple births, LBW, and preterm births were also only recorded in 2016 relative to 2012.

**Table 2:** Comparison of delivery mode and pregnancy outcomes over time

Variable		2012 (***N*** = 3480)		2014 (***N*** = 4044)		2016 (***N*** = 4194)		OR1 and 95% CI	OR2 and 95% CI
		***n***	%	***n***	%	***n***	%		
*For all childbearing women*									
Delivery mode	Vaginal	1962	56.38	2424	59.94	2444	58.27	**0.86 (0.79–0.95)**	0.93 (0.85–1.01)
	CS	1518	43.62	1620	40.06	1750	41.73		
Vaginal delivery	Instrumental	75	3.82	130	5.36	120	4.91	**1.43 (1.07–1.91)**	1.30 (0.98–1.75)
	Natural	1887	96.18	2294	94.64	2324	95.09		
CS	Emergency	719	47.36	903	55.74	690	39.43	**1.40 (1.22–1.61)**	**0.72 (0.63–0.83)**
	Elective	799	52.64	717	44.26	1060	60.57		
Pregnancy complications		854	24.54	1062	26.26	1326	31.62	1.10 (0.99–1.22)	**1.42 (1.29–1.57)**
Multiple births		71	2.04	102	2.52	211	5.03	1.15 (0.81–1.62)	**2.92 (2.19–3.89)**
Stillbirth		63	1.81	35	0.82	41	0.98	**0.47 (0.31–0.72)**	**0.54 (0.36–0.80)**
LBW		239	6.87	297	7.34	424	10.11	1.08 (0.90–1.28)	**1.53 (1.29–1.80)**
Preterm birth		349	10.03	390	9.64	570	13.59	0.96 (0.82–1.12)	**1.41 (1.23–1.63)**
BDs		59	1.70	33	0.82	41	0.98	**0.48 (0.31–0.73)**	**0.57 (0.38–0.85)**
Missing		18	/	11	/	0	/		
Sex of infant	Girl	1647	47.33	1950	48.22	2017	48.09	1.03 (0.94–1.13)	1.03 (0.94–1.13)
	Boy	1817	52.21	2086	51.58	2164	51.60		
	Missing	16	/	8	/	13	/		
*For primipara (parity < 1)*									
Delivery mode	Vaginal	373	44.40	659	45.42	1006	48.83	0.96 (0.81–1.14)	**0.84 (0.71–0.98)**
	CS	467	55.60	792	54.58	1054	51.17		
Vaginal delivery	Instrumental	8	2.14	11	1.67	20	1.99	0.77 (0.31–1.94)	0.93 (0.40–2.21)
	Natural	365	97.86	648	98.33	986	98.01		
CS	Emergency	195	41.76	372	46.97	345	32.37	1.24 (0.98–1.56)	**0.68 (0.54–0.85)**
	Elective	272	58.24	420	53.03	709	67.27		
Pregnancy complications		158	18.81	277	19.09	551	26.75	1.02 (0.82–1.27)	**1.58 (1.29–1.92)**
Multiple births		24	2.86	31	2.14	67	3.25	0.74 (0.43–1.27)	1.14 (0.71–1.84)
Stillbirth		21	2.50	16	1.10	20	0.97	**0.44 (0.23–0.84)**	**0.38 (0.21–0.71)**
LBW		77	9.17	113	7.79	192	9.32	0.84 (0.62–1.13)	1.02 (0.77–1.34)
Preterm birth		113	13.45	167	11.51	277	13.45	0.84 (0.65–1.08)	1.00 (0.79–1.26)
BDs		14	1.67	14	0.96	19	0.92	0.57 (0.27–1.20)	0.55 (0.27–1.09)
Missing		7	0.83	5	0.34	0	/		
Sex of infant	Girl	390	46.43	694	47.83	942	45.73	1.05 (0.89–1.25)	0.97 (0.82–1.14)
	Boy	445	52.98	754	51.96	1110	53.88		
	Missing	5	0.60	3	0.21	8	0.39		
*For multipara (parity ≥ 1)*									
Delivery mode	Vaginal	1589	60.19	1765	68.07	1438	67.39	**0.71 (0.63–0.80)**	**0.73 (0.65–0.83)**
	CS	1051	39.81	828	31.93	696	32.61		
Vaginal delivery	Instrumental	67	4.22	119	6.74	100	6.95	**1.64 (1.21–2.23)**	**1.70 (1.24–2.33)**
	Natural	1522	95.78	1646	93.26	1338	93.05		
CS	Emergency	524	49.86	531	64.13	345	49.57	**1.80 (1.49–2.17)**	0.99 (0.82–1.20)
	Elective	527	50.14	297	35.87	351	50.43		
Pregnancy complications		696	26.36	785	30.27	775	36.32	**1.21 (1.08–1.37)**	**1.59 (1.41–1.80)**
Multiple births		47	1.78	71	2.74	144	6.75	**1.55 (1.07–2.25)**	**3.99 (2.86–5.58)**
Stillbirth		42	1.59	19	0.73	21	0.98	**0.46 (0.27–0.79)**	0.62 (0.36–1.04)
LBW		162	6.14	184	7.10	232	10.87	1.17 (0.94–1.45)	**1.87 (1.51–2.30)**
Preterm birth		236	8.94	223	8.60	293	13.73	0.96 (0.79–1.16)	**1.62 (1.35–1.95)**
BDs		45	1.70	19	0.73	22	1.03	**0.42 (0.25–0.73)**	0.60 (0.36–1.00)
Missing		11	0.42	6	0.23	0	/		
Sex of infant	Girl	1257	47.61	1256	48.44	1075	50.37	1.03 (0.92–1.15)	1.11 (0.99–1.25)
	Boy	1372	51.97	1332	51.37	1054	49.39		
	Missing	11	0.42	5	0.19	5	0.23		

Using stratified analysis, primipara were more likely to have had a higher rate of pregnancy complications in 2016 compared to 2012. In regard to CS, the percentages of overall CS and emergency CS decreased in 2016 relative to 2012, and stillbirth rates dropped dramatically throughout the study period.

Regarding multipara, the proportions of women who underwent vaginal delivery and instrumental vaginal delivery were more common in 2014 and 2016 than in 2012. The emergency CS rate only increased significantly in 2014 compared with 2012, and the proportions of pregnancy complications and multiple births significantly rose in 2014 and 2016 relative to 2012. Stillbirth and BDs were less likely to be reported in 2014 than in 2012, and LBW and preterm birth risks increased only in 2016 compared to 2012.

### Multiple analysis of main factors with policy changes

Adjusting for confounders such as maternal age, pregnancy predictions, multiple births, birth weight, hospital level, CS history, birth week, and maternal parity, CS risk decreased significantly in 2014 (OR_adj_ = 0.62; 95% CI, 0.55–0.67) relative to 2012. In addition, we only observed elevated risks of pregnancy complications (OR_adj_ = 2.30; 95% CI, 1.86–2.83) and multiple births (OR_adj_ = 3.25; 95% CI, 2.19–4.83) in 2016 compared to 2012, when maternal age, birth weight, birth week, hospital level, delivery mode, and maternal parity were added to the adjustments; risks of LBW/ preterm birth turned out be non-statistic significance in 2014 (OR_adj_ = 0.95; 95% CI, 0.81–1.10) and 2016 (OR_adj_ = 0.90; 95% CI, 0.70–1.15) compared to 2012 in adjusting mode, considering maternal age, multiple births, pregnancy complications, hospital level, delivery mode, and maternal parity.

## Discussion

The “one-child” policy in modern China has profoundly affected the lives of approximately one-fifth of the world’s population for nearly 35 years [1]. Following the change in birth policy from “one-child,” to “second-child,” to “universal second-child,” we observed (1) an increased proportion of childbearing women of advanced reproductive age, with better education, a greater number of multipara, repeated CS at enrollment; (2) that births at a university/(tertiary) hospital and by vaginal delivery increased over time; (3) and after adjusting for confounders, that the risk of CS decreased; however, risks of pregnancy complications and multiple births were elevated, especially after the “universal second-child” policy was instituted and promulgated.

The results of the present study revealed an upward trend in the proportion of women with advanced age over the years analyzed. In 2016, 14.47% women who gave birth were 35 years or over, which was more than double the figure in 2012, and this was consistent with our expectations as delayed childbearing has become prevalent globally [6–9]. In the USA, the mean maternal age exceeded 35 years, accounting for 14.0% of the total number of singleton births in a single center study [7]. Over 10% of nulliparous women at first delivery were aged 35 years or more according to Finnish national data [9]; meanwhile, in Germany, the corresponding proportion at advanced maternal age was as high as 22% [10]. In China, the latest national percentage of mothers over 35 years of age in 2007 was 8.56% [11]. By stratified analysis, this index was significantly augmented among women who gave birth, which for the most part supported the hypothesis by the new policy’s framers that entitled older women to have additional children.

The increase in the proportion of women with improved education agreed with a large-sample survey in China that revealed that highly educated women were strongly in favor of raising a second child [12]. Higher education might also bring higher income, making a plan for two babies more affordable and realistic, as was reported in Europe [13]. Moreover, the majority of women with inadequate education came from rural areas, where the “one-child” policy was not so strictly enforced; therefore, the changes in birth policy might not greatly affect their desire for additional children. The relationship between policy transfer and body mass index categories was also positive. The improved education of childbearing women might additionally contribute to healthier lifestyle and better control of their weight.

These results generally suggest a high CS rate and previous CS for all women, and might reflect influences of the rise in CS for all socioeconomic regions in China [14]. Our CS rate of approximately 40% was far higher than the global level (18.6%) based on the latest data from 150 countries but less than the overall CS rate (54.9%) in mainland China in 2011, based on a multicenter and large-sample study [15, 16]. Notably, the CS rate rose above 50% among women who gave birth, which was generally much higher than the corresponding data for Taiwan (26.0%), Germany (24.0%), and younger childbearing women in Columbia (36.9%) but approximately the same as for data from Iran (44.6–53.1%) [10, 17–19]. Before the introduction of the “second-child” policy, the maternal request for CS was the commonest indicator for a cesarean in China [16]. A previous CS might be one of the primary indicators for a future CS, therefore, we also noticed a relative high CS rate in 2016 in our study than other researches currently. Fortunately, decreased risk of CS has been observed with policy changes, even after adjusting for confounders. The Zhejiang government and institutions have also made great strides over recent years in integrating intervention into enhancing antenatal care and avoiding CS, particularly unnecessary CS.

The results of the present survey revealed an increasing number of deliveries at a university hospital over the years analyzed. On the one hand, awareness of healthcare led more women to seek care at high-level hospitals. On the other hand, a rising proportion of pregnancy complications was a more critical determinant, which brought women to seek further medical care. In our study, the rising risks of pregnancy complications highlighted our attention, particularly with respect to the impacts of confounders upon multiple regression model analysis. This might arise from a combination of advanced maternal age, repeated CS, greater numbers of multiparous woman and multiple births, as documented previously [6, 9, 20–24]. In Victoria, Australia, older women giving birth—regardless of whether they were nulliparous or multiparous—were at higher risk of obstetric morbidities, with odds ratios ranging from 1.58 to 2.44 [9]. In China, data from 39 hospitals indicated that women over 35 years manifested a higher risk of a wide variety of pregnancy complications compared to women aged 25–29 years [20]. Women with CS easily develop major pregnancy-associated diseases (e.g., preeclampsia, gestational hypertension, and hemorrhage) in a subsequent pregnancy relative to women who deliver vaginally [21, 22]. In addition, higher gravidity and parity correlated positively with coronary heart disease, metabolic syndrome, and CS [23, 24]. The most frequently presented complications of pregnancy in the current study were premature rupture of membranes, gestational hypertension, gestational diabetic mellitus, and severe anemia; in turn, these factors might increase the risk of LBW and preterm birth. Fortunately, risk of LBW /preterm birth did not increase after adjusting for confounders. Due to technologic developments in antenatal screening and neonatal treatments, the incidence of stillbirth and BDs did not worsen with the modifications to China’s fertility policy. Since the beginning of “second-child” policy, we offered special outpatient services for women with advanced age, repeated CS, with desire for second child in Zhejiang province. We strengthened the management of women with high pregnancy risk factors. All above mentioned measures helped to improve pregnancy outcomes to some extent.

### Strengths and limitations

The principal strength of the current study was the evaluation of the alterations in the socio-demographic characteristics and outcomes for pregnant women before and after the end of the one-child policy in China, with the adjustment in reproductive policy receiving much interest in China. A study based on three different hospitals ranging from a tertiary hospital to a primary hospital’s multicenter study of childbearing women significantly reduced overall selection bias. We then performed the stratified and multiple regression model analysis to avoid confounding factors.

Some limitations should be acknowledged when interpreting our results. First, there were no detailed data on women with early adverse pregnancy outcomes such as miscarriage, as well as some economic characteristics. Thus, we could not make a calculated estimate of all pregnancy outcomes, and other possible impacts. In terms of economic characteristics, basic antenatal health care services and most prenatal screening for BDs are free in Zhejiang. Therefore, pressures from economy for women were minor actually. Second, China is a country with large economic and healthcare disparities. As the data were based primarily on women living on the eastern coast of China, the results could only provide a reference, and might not be extrapolated to the general population. Finally, we could not identify direct causal relationship with respect to desire to conceive, birth behaviors, and birth outcomes among these women, as we were limited by the data. As we know, socio-demographic factors were also strongly associated with pregnancy outcomes, and both of them could be affected by birth policies. Our findings thus only reflected some alterations in maternal characteristics and outcomes related to birth-policy modifications in a short term period.

## Conclusions

The present study indicated some differences in maternal characteristics after ending China’s “one-child” policy. It was significant to be aware of the possible impacts on delivery mode and pregnancy outcomes. We observed increased births at a tertiary hospital and by vaginal delivery, and an increase in some critical characteristics of childbearing women—including advanced age, improved education, multipara and more repeated CS thought out the periods. We also found increased risks of pregnancy complications and multiple births. In consideration of these findings, effective public health strategies targeting certain women should be advocated.

## Data Availability

The datasets are available from the corresponding author upon reasonable request.

## Abbreviations

BMI: Body mass index
OR: Odds ratio
CI: Confidence intervals
LBW: Low birth weight
CS: Cesarean section
BDs: Birth defects
